# HPLC and CE Procedures for the Determination of Fluoroquinolones (2020–2025)

**DOI:** 10.3390/molecules31040651

**Published:** 2026-02-13

**Authors:** Paweł Kubalczyk, Izabella Kośka, Rafał Głowacki

**Affiliations:** Department of Environmental Chemistry, Faculty of Chemistry, University of Lodz, 163/165 Pomorska Str., 90-236 Łódź, Poland; iza.k755@gmail.com (I.K.); rafal.glowacki@chemia.uni.lodz.pl (R.G.)

**Keywords:** CE, determination, fluorescence, fluoroquinolones, HPLC, mass spectrometry, UV-Vis

## Abstract

Fluoroquinolones (FQLs) are synthetic derivatives of the quinolone class, originally developed from the nalidixic acid scaffold. FQLs are widely used in human and animal medicine due to their broad spectrum of activity against Gram-positive and Gram-negative bacteria. Their strong bactericidal properties result from the inhibition of bacterial DNA transcription and replication. However, inappropriate use of these antibiotics often leads to toxic side effects, environmental pollution, and the development of antibiotic resistance in bacteria. Recently, numerous methods for determination of FQLs in various matrices have been developed using separation techniques such as high-performance liquid chromatography and capillary electrophoresis. In recent years, analytical procedures have employed a range of detection methods, including UV–Vis spectrophotometry, electrochemiluminescence, mass spectrometry, and fluorescence. Most of these procedures involve some form of extraction during the sample preparation step. This report summarizes the development of FQL determination methods over the last five years and may assist in selecting an appropriate procedure for a given sample matrix.

## 1. Introduction

Fluoroquinolones (FQLs) are compounds derived from nalidixic acid [1]. They are a group of synthetic antibiotics often used in the treatment of humans and animals. They are very willingly administered to animals by breeders due to their universality, as they are characterized by a wide spectrum of activity against both Gram-positive and Gram-negative bacteria [1].

They owe their strong bactericidal properties to the ability to inhibit the transcription and replication of bacterial DNA. These compounds are used to treat respiratory diseases, urinary tract infections, and stomach disorders. Moreover, they have excellent bioavailability thanks to rapid absorption after oral administration and a broad spectrum of action.

**Figure 1:**
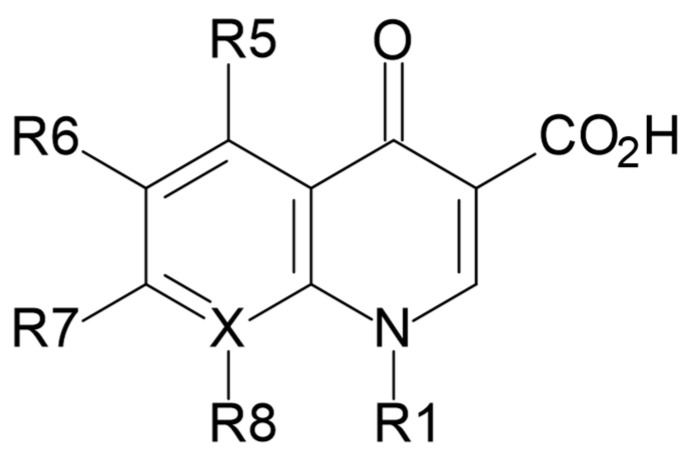
Quinolone core structure.

Quinolones are a family of antibiotics containing a bicyclic core linked to the 4-quinolone compound (Figure 1) [1,2].

Since their discovery in the early 1960s, they have gained importance as key compounds in the treatment of both community-acquired and severe nosocomial infections [3]. Nalidixic acid is generally considered to be the first quinolone antibiotic, a drug with a narrow spectrum of activity against intestinal bacteria, used in the treatment of uncomplicated urinary tract infections [2,4].

In the 1970s, there was a breakthrough in the importance of this class of compounds thanks to the development of FQLs, which show a much broader spectrum of action, but also much better pharmacokinetics compared to first-generation quinolones, less binding to proteins, higher drug tolerance, lower toxicity and a longer half-life [5,6]. Ciprofloxacin (CIP) and ofloxacin (OFL) can be distinguished as representatives of this group of antibiotics. They are active against both Gram-negative and Gram-positive pathogens, and importantly, they are also active against the causative agent of tuberculosis, *Mycobacterium tuberculosis* [2].

The main problem associated with the use of these compounds is the fact that antibiotics from this group are administered to sick animals not only for therapeutic purposes, but also prophylactically to avoid disease among entire herds. These chemical compounds, in addition to their intended pharmacological effects, are often characterized by undesirable or even toxic effects that are dangerous to humans, and the excretion of residues of these compounds in urine and feces leads to environmental pollution.

Giving animals antibiotics without a clear reason and not observing the withdrawal period leads to the development of antibiotic-resistant bacteria. The occurrence of bacteria resistant to antibiotics is called antibiotic resistance. Infections caused by antibiotic-resistant bacteria are very difficult or even impossible to cure. Their use carries a huge risk for humans, because they may cause allergic or toxic reactions in hypersensitive people. Antibiotic residues also increase the risk of fungal diseases and the emergence and increase in microbial resistance to these antibiotics.

The administration of veterinary drugs should be stopped for an appropriate period before the slaughter of animals, as eating meat with residues of such drugs is also dangerous to the health of consumers.

## 2. FQL Activity

The activity of first-generation quinolones was limited only to Gram-negative organisms, excluding Pseudomonas species [7]. The first second-generation quinolone, flumequine (FLU), is an example that adding a fluorine atom at the R6 position can significantly improve the activity spectrum [8]. This change in the structure of the molecule allows it to radically increase the activity of quinolones. Almost all antibiotics defined as second-generation fluoroquinolones, e.g., enoxacin (ENO), norfloxacin (NOR) and ciprofloxacin (CIP), can inhibit all Gram-negative organisms, including Pseudomonas species [9].

After adding a fluorine substituent, these drugs were further modified by adding a piperazine ring to the R7 position and adding a cyclopropyl group to the R1 position. The R7 piperazine ring improved potency against Gram-negative bacteria, while the cyclopropyl group improved the overall activity of the compounds, increasing their potency [10,11].

This combination in the structure of the CIP molecule made it the most active compound among the early second-generation compounds and made it the first choice currently used against Pseudomonas aeruginosa [12]. Subsequent modifications of the structure of second-generation FQLs molecules, leading to OFL, enabled the inhibition of the activity of Gram-positive organisms [13]. Of all the compounds belonging to this group, OFL is considered the most powerful because it contains all new substituents and is therefore still used in clinical treatment today.

## 3. Pharmacokinetics, Pharmacodynamics

The development of quinolones in terms of pharmacokinetics and pharmacodynamics involves improving antibiotic dosing strategies to increase their effectiveness and prevent bacterial mutations to increase resistance. The first quinolone compound, nalidixic acid, has been limited in use due to low serum levels. However, modifications to the structure of this compound by introducing substituents in later generations of quinolones led to improved oral absorption [1]. These modifications also resulted in an increase in half-life that allowed once-daily dosing of some second-generation agents and all later-generation agents.

Over time, changes in the metabolism of quinolones have been observed, with earlier generations of these compounds being primarily eliminated from the body by renal metabolism and clearance, while later quinolones have been modified to become agents with non-renal clearance. In summary, quinolones exhibit concentration-dependent bactericidal activity with a persistent post-antibiotic effect. Key structural modifications to improve quinolone pharmacokinetics were based on modifications at the R5, R6, R7, and R8 positions (Figure 1), resulting in longer half-life, improved tissue penetration, and improved bioavailability. The addition of an amino group at the R5 position increased the lipophilicity of quinolones, while the fluorine substituent at the R6 position turned out to facilitate penetration into the bacterial cell [6,14]. The addition of substituents at the R7 position mediated the improvement of the half-life and penetration of bacterial tissues. The azabicyclic group and the piperazine group at the R7 position extended the half-life of the agents to over 10 h by further increasing the lipophilicity [15,16].

Another substituent at this position are pyrrolidine rings, but this modification is crucial to enhance the potency of quinolones. It has been reported to be associated with unfavorable water solubility and oral bioavailability. To improve these physical properties, subsequent generations of quinolones introduced a methyl group into the rings. Furthermore, alkylation of the rings at the R7 position increased the half-life and bioavailability of the agents. The addition of this group to the piperazine rings significantly extended the half-life of OFL compared to CIP, which has only a piperazine group in its structure [17,18].

The latest research on key modifications of quinolone structures leads to inhibiting the development of bacterial resistance to quinolones.

## 4. Clinical Indications for the Use of Fluoroquinolones and Their Toxicology

Quinolones are broad-spectrum antibiotics with many clinical indications. In recent years, FQLs have been the main drugs used to treat urinary tract infections and infections of the gastrointestinal and respiratory tracts [1]. Unfortunately, overprescription of quinolones by physicians has led to the rapid development of bacterial resistance to these antibiotics, which in turn leads to the drugs losing their effectiveness [19].

The most common side effects reported following quinolone administration are gastrointestinal disturbances and/or arthralgia. Due to concerns associated with this, this class of compounds is restricted to prescription to pediatric patients [20]. Another side effect is phototoxicity, caused by the accumulation of sensitive drugs in the skin, where they can be activated by exposure to sunlight, causing skin damage [21]. Some quinolones have also caused central nervous system reactions, including dizziness, insomnia, and headache. This side effect has been shown to be related to the inhibition of GABA receptors, the primary inhibitory neurotransmitter, and has been observed following administration of agents with an additional effect of the R7 group [22].

## 5. Physical Properties of FQLs

Fluoroquinolones are among the most frequently used antibiotics. They are used in both human and veterinary medicine worldwide. Fluoroquinolones exhibit a broad spectrum of activity, high efficacy against Gram-negative and Gram-positive bacteria, and good bioavailability, which is why they have gained widespread use in the treatment of various infections. In clinical settings, they are used for the treatment of respiratory, urinary, gastrointestinal, and soft tissue infections, among others [1]. In veterinary medicine, fluoroquinolones play a key role in the treatment of infectious diseases in farm animals and companion animals, which impacts both animal health and the safety of food of animal origin [6]. This chapter will present the most used active substances from the fluoroquinolone group and a brief description of them. In humans, CIP, OFL and LEV are the most common, while in animals, other FQLs predominate, such as ENR, SAR, and similar ones—often specific to the type of animal. Some of these compounds are briefly characterized below.

### 5.1. Ciprofloxacin

**Figure 2:**
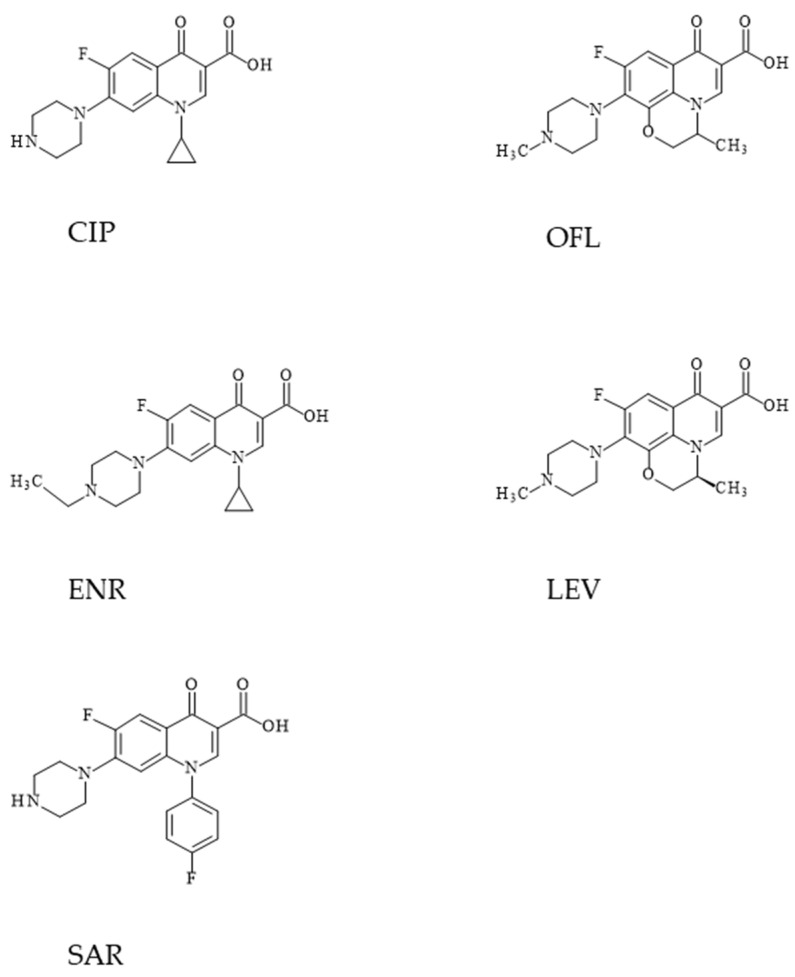
Structures of selected fluoroquinolones.

1-cyclopropyl-6-fluoro-4-oxo-7-piperazin-1-ylquinoline-3-carboxylic acid (CIP) is a quinolone compound belonging to the second generation of FQLs, having in its structure cyclopropyl, carboxylic acid, fluorine atom and piperazin-1-yl in positions 1, 3, 6 and 7, respectively. The structural formula of CIP is shown in Figure 2. Commercially, CIP is available in the form of a crystalline powder with a color ranging from white to light yellow, slightly soluble in water, but highly soluble in 0.1 mol/L HCl [14]. The pH of aqueous solutions of CIP hydrochloride is 1.5–7.5 and these solutions are stable for at least 14 days at room temperature [23]. The pKa value of the carboxyl group of the CIP molecule is 6.09, while the pKa value of the nitrogen atom in the piperazinyl ring is 8.74 [24].

### 5.2. Ofloxacin

(±)-9-fluoro-2,3-dihydro-3-methyl-10-(4-methyl-1-piperazinyl)-7-oxo-7H-pyridol[1,2,3-de]-1,4-benzoxazine-6-carboxylic acid (OFL) is an oxazinoquinolone containing carboxyl, fluorine, methyl and 4-methylpiperazine. The structural formula of OFL is shown in Figure 2.

This organic heterocyclic compound is a white to light yellow crystalline powder. OFL is soluble in aqueous solutions with a pH between 2 and 5 and above 9. However, it is slightly soluble in aqueous solutions with a pH 7 (the solubility in such solutions drops to 4 mg/mL) [25]. The pKa value of the carboxyl group of the OFL molecule is 5.97, while the pKa value determined by the presence of the piperazinyl ring is 9.28 [26].

### 5.3. Sarafloxacin

6-fluoro-1-(4-fluorophenyl)-1,4-dihydro-4-oxo-7-(1-piperazinyl)-3-quinolinecarboxylic acid (SAR) is a chemical compound belonging to the fluoroquinolone class with a specific chemical structure, used in animals as an antibacterial drug. The structural formula of SAR is shown in Figure 2. This drug has not been approved for human use in the US and Canada. As a fluoroquinolone, it works by inhibiting bacterial enzymes responsible for the synthesis and structure of bacterial DNA, resulting in inhibition of bacterial cell division and cell death. It is an inhibitor of the CYP1A2 enzyme (cytochrome P450 1A2). EU regulations specify that the SAR residue limit in the liver of chickens is 100 µg/kg, and for salmonid fish, 30 µg/kg [25].

### 5.4. Enrofloxacin

1-cyclopropyl-7-(4-ethyl-1-piperazinyl)-6-fluoro-1,4-dihydro-4-oxo-3-quinolinecarboxylic acid (ENR) is an effective broad-spectrum fluoroquinolone antibiotic intended for animals. The drug is used exclusively in veterinary medicine and is intended for the treatment of infections of the respiratory, gastrointestinal, and urogenital tracts in animals such as cattle, pigs, poultry, dogs, and cats. It has bactericidal activity, primarily against Gram-negative and partially Gram-positive bacteria, by inhibiting enzymes responsible for DNA synthesis in bacteria. According to the European Commission, the MRL for enrofloxacin in tissues of various animal species ranges from 100 µg/kg to 300 µg/kg [25]. The structural formula of ENR is shown in Figure 2.

### 5.5. Levofloxacin

(S)-9-fluoro-2,3-dihydro-3-methyl-10-(4-methylpiperazin-1-yl)-7-oxo-7H-pyrido[1,2,3-de]-1,4-benzoxazine-6-carboxylic acid (LEV) is a fluoroquinolone antibiotic and the optical S-(−) isomer of racemic OFL. LEV belongs to the third generation of fluoroquinolones used against Gram-positive bacteria in respiratory infections. Commercially, LEV is available in the form of a crystalline, light yellowish powder, freely soluble in glacial acetic acid or chloroform, and sparingly soluble in water [27].

## 6. Methods for the Determination of Fluoroquinolones

A literature search was performed in the SCOPUS^®^ and Web of Science^™^ databases in June 2025. The search query, applied to titles, abstracts, and keywords, was formulated as follows: (antibiotics) AND (fluoroquinolones) AND (determination OR detection). The search was restricted to publications from the last five years (2020–2025). The review focused on a specific class of antibiotics (fluoroquinolones) widely used in human and veterinary medicine, with particular emphasis on analytical techniques, sample preparation procedures, and detection methods. Based on these criteria, 127 records were initially identified. Further refinement limited the results to studies employing HPLC or CE techniques. Articles were included if they reported an analytical method, described sample preparation, specified the matrix, and provided data on the limit of detection and/or limit of quantification and/or linear range. Following this screening, a total of 82 articles were selected for analysis.

Some antibiotics, including FQLs, are increasingly used in animal husbandry due to the need for their rapid growth and to avoid disease among entire herds [26,28]. Environmental pollution occurs, among other factors, because of animal urine and feces containing residues of these substances. At the same time, their application constitutes a potential threat to human health. Continuous exposure of humans and ecosystems to antibiotic residues contributes to the emergence of antibiotic resistance. This is a highly adverse phenomenon, as infections caused by resistant bacterial strains are often extremely difficult, and in some cases impossible, to cure [28].

Due to the establishment of MRLs for veterinary antibiotics in food of animal origin [28], laboratories are forced to develop increasingly simpler, faster and more precise methods for determining antibiotic residues in meat and the environment.

Numerous separation methods for determination of FQLs in last 5 years were published. These procedures are predominantly based on HPLC [29,30,31,32,33,34,35,36,37,38,39,40,41,42,43,44,45,46,47,48,49,50,51,52,53,54,55,56,57,58,59,60,61,62,63,64,65,66,67,68,69,70,71,72,73,74,75], but also on CE [76,77,78,79,80,81,82] techniques. Analytical procedures using CE were based on electrochemiluminescent [76,77,78] or spectrophotometric detection [79,80,81,82], while HPLC methods utilize fluorescence [29,30,31,32,33,34,35,36,37], mass spectrometry [38,39,40,41,42,43,44,45,46,47,48,49,50,51,52,53,54,55,56,57,58] and UV-Vis detection [32,59,60,61,62,63,64,65,66,67,68,69,70,71,72,73,74,75]. A concise summary of these procedures, along with some analytical parameters, is provided in Table 1 below.

Each analytical procedure consists of several steps, including collection, transport, storage, sample preparation, separation and detection of sample components, and analysis of the obtained results. Each of these steps is important, but due to the potential for errors, sample preparation seems to be the most important. It is worth emphasizing that sample preparation is a crucial step in the analytical process, particularly when analyzing complex matrices or analytes present at very low concentrations, where high sensitivity is required. The purpose of this step is to minimize matrix interferences while simultaneously extracting, purifying, and preconcentrating the analyte. Since fluoroquinolones (FQLs), aside from pharmaceutical formulations, are usually found at trace levels, preconcentration is essential to achieve reliable quantitative results. Samples were prepared in various ways, depending directly on the type/state of the sample. Aqueous samples (environmental water, river water, medications, eye or ear drops) were the easiest to prepare, usually requiring only appropriate dilution or preconcentration. The most demanding matrices are biological samples (plasma, urine, meat, eggs), known for their complexity, because they may contain components that must be removed before the sample is introduced into the separation system (e.g., proteins in plasma), or require prior grinding/homogenization (e.g., hard tablets, meat).

### 6.1. CE Methods

Recently, seven electrophoretic methods for determining FQLs in various matrices have been developed. These methods utilize different mechanisms to efficiently separate sample components. Capillary zone electrophoresis [78,80,81,82], micellar electrokinetic chromatography [79], and cyclodextrin electrokinetic chromatography [76,77] were used for sample separation and analytes detection. The most used separation buffers were phosphate solutions [76,77,78,79] or a mixture of phosphates and borates [80,81,82], with concentrations ranging from 0.02 to 0.1 mol/L, with optional additions of sodium dodecyl sulfate [79] or cyclodextrins [76,77]. CE procedures use either UV [79,80,81,82] or ECL [76,77,78] detection.

All CE-ECL methods used electrochemiluminescent detection based on tris (2,2′-bipyridyl) ruthenium (II) (Ru(bpy)_3_^2+^) which is very attractive analytical method for organic amines due to its high sensitivity and selectivity [76]. It is widely recognized that the luminescence intensity of (Ru(bpy)_3_^2+^) can be greatly increased by compounds containing amino group in their molecular structure [83]. Fortunately, each fluoroquinolone contains at least two tertiary amino groups, what results in significant enhancement of detector signal [78].

The other CE methods, devoted to FQL determination in food or human urine, use spectrophotometric detection in the UV range, with analytical wavelengths set to 214 nm, 288 nm, 271 nm, and 285 nm [79,80,81,82].

### 6.2. HPLC Methods

As mentioned earlier, HPLC-based methods use three types of detection, namely FL, MS, and UV. In the case of methods using FL detectors, a necessary condition is that the compounds being determined are capable of fluorescence or have been previously subjected to chemical derivatization, resulting in fluorescent derivatives. FQLs naturally exhibit high fluorescence; thus, the use of HPLC with FL detection is a logical solution. However, since FQLs occur in the environment in small or even trace amounts, despite the very good sensitivity of FL detectors, it is necessary to use an efficient analyte concentration method during sample preparation [30].

A comparable situation is observed when UV–Vis spectrophotometry is employed as the detection technique. In this case, the analyte must inherently possess a chromophore within its molecular structure. Similar to FL detection, suitable optical properties can also be introduced through chemical derivatization. However, among the studies discussed, all those utilizing UV detection relied exclusively on the intrinsic absorption characteristics of FQLs.

Interestingly, although it is generally believed that analytical methods using highly sensitive detectors such as fluorescence, EL or MS allow for achieving lower LOD and LOQ, the comparison shows that procedures which use UV detection are competitive in this respect. This is due to the sample preparation process using very efficient extraction methods. These highly selective methods enable substantial simplification of the sample matrix while simultaneously concentrating the analytes.

In recent years, numerous HPLC-MS methods have been described for the determination of FQLs in plasma [38,39,45,53], food [41,42,43,44,46,51,52,54,55,56], and aqueous samples [40,47,48,49,57,58]. Mass detectors, considered extremely sensitive and versatile, enable obtaining additional information on the structure of the analyzed compounds. However, the most important reasons for the limited use of HPLC-MS equipment in clinical laboratories are the high purchase and operating costs.

The C-18 reversed phase is the most used in chromatographic analysis of FQLs because its hydrophobic (nonpolar) surface strongly interacts with FQLs, which are moderately polar compounds, enabling effective separation of their mixtures. Octadecylsilane chains on the stationary phase surface provide strong retention and selectivity for these compounds, and this phase is characterized by high chemical stability and wide pH tolerance (usually in the range of approximately 2 to 8). Using an acidic mobile phase (e.g., with a pH of approximately 2–3) is crucial because, under such conditions, fluoroquinolones exist primarily in a less ionized form, which enhances their retention and stabilizes their chromatographic behavior, leading to more reproducible analytical results. Acidic pH also minimizes the dissociation of functional groups, thereby reducing electrostatic interactions and promoting more uniform retention based on hydrophobic interactions with the C-18 phase. Choosing an acidic pH stabilizes the equilibrium between the chemical forms of FQLs, allowing for more efficient separation and the appearance of sharper, better-defined chromatographic peaks.

In the vast majority of chromatographic procedures, regardless of the sample matrix, compound separation was performed in gradient elution mode [29,30,31,32,33,35,36,38,39,40,41,42,43,44,45,46,49,50,51,54,55,56,57,58,61,62,66,67,75] on columns packed with a C-18 stationary phase [29,30,32,34,35,37,39,40,41,42,43,44,46,48,49,51,53,54,55,56,57,58,59,60,61,62,63,64,65,66,67,68,69,70,71,72,73,74,75].

In HPLC techniques employing columns with small or very small particle sizes, ACN is generally preferred to MeOH. As a stronger eluent, ACN helps preserve the HPLC system’s performance because ACN/water mixtures produce lower back pressures [31].

It is worth noting that, consistent with the principles of green chemistry, recent advances in chromatographic method development focus on minimizing or eliminating the use of buffer systems [34]. The use of ammonium salts (such as nitrate, acetate, or formate) in the mobile phase plays a crucial role in optimizing both chromatographic and mass spectrometric performance in HPLC-MS analyses. These volatile buffers enhance ionization efficiency by promoting consistent proton transfer and mitigating ion suppression effects during electrospray ionization. As a result, improved signal intensity, analytical precision, and overall method reproducibility are achieved. Furthermore, ammonium-based additives ensure compatibility with mass spectrometric detection by preventing the accumulation of non-volatile residues and maintaining stable spray conditions, which is particularly important for long analytical sequences.

In addition to buffering effects, the incorporation of formic acid into the mobile phase contributes significantly to analytical performance by lowering the pH and facilitating the ionization of analytes in positive ESI mode. The acidic environment not only enhances ion formation but also improves the stability and reproducibility of mass spectrometric responses. Moreover, formic acid can influence chromatographic behavior by modulating the ionization state of functional groups and altering the surface charge distribution of the stationary phase. These combined effects lead to more efficient separation, improved peak symmetry, and enhanced overall chromatographic resolution. We can note this trend in many of the procedures discussed in this review. 0.1% formic acid is often added to the mobile phase (water and ACN) to improve separation and enhance the signal in MS detection [71]. In fact, most discussed here MS procedures [38,39,40,41,42,43,44,45,46,48,49,51,52,53,54,55,56,57,58] and some others [30,32,63,67,75] utilize this additive. In other procedures, phosphates of different pH were readily used [29,31,33,37,59,60,61,64,65,66,68,69,70,71,72,73,74].

### 6.3. Matrices

Among the CE techniques, the analyzed samples included fish (carp, eel) [76,77,78], meat (liver, kidney) [80,81], biological fluids (human urine) [82], and liquid samples (milk, honey, water) [79]. HPLC procedures have been applied to the analysis of environmental liquid samples (water, sewage) [29,30,36,37,40,47,49,57,58,64,65,72], food (meat, milk, honey) [33,35,41,42,43,44,46,50,51,52,54,55,56,61,63,67,68], biological, human and animal samples (urine, plasma, aqueous humor) [31,32,34,38,39,45,48,53,60,75], and drugs [59,62,66,69,70,71,73,74].

Reviewing the publications, one can easily conclude that most sample preparation procedures utilize some type of extraction. These include extraction or microextraction using ACN [31,42,52,56,70], ACN/MeOH mixture [32,39,43,45], MeOH [55,72], dichloromethane/ACN and dichloromethane/chloroform [81,82], SPE and SPME [40,46,47,56,57,58,60,75,77,80], QuEChERS extraction [49,72,78], extraction using modified nanoparticles [61,63,65,79,80], DLLME [37,51,65], and metal–organic frameworks [30,41,68].

Microextraction methods, including SPE, are widely used in FQL determination because they offer significant advantages over traditional liquid–liquid extraction. These include the excellent adsorption capacity of the materials used, the possibility of partial automation of the analytical procedure, which reduces errors, improves method repeatability and precision, and significantly reduces sample and eluent consumption. Furthermore, these methods allow for increased selectivity and sensitivity [30].

Among the described extraction procedures, various solvents were used with the aim of adjusting pH of the sample. This step was necessary because FQLs exhibit amphoteric acid-base behavior. This is a direct consequence of the presence of both carboxyl and protonated amino group on the piperazine ring, which influences their chemical properties and interaction mechanisms. The pKa values of most fluoroquinolones fall within the range of 4.4–5.8 for the carboxyl group and 7.3–9.5 for the amino group on the piperazine ring. Under such conditions, the carboxyl group remains predominantly in its non-ionized form, whereas the piperazine nitrogen is protonated. This specific protonation state enhances the analyte’s affinity toward the organic phase, leading to an increased partition coefficient and improved extraction efficiency. Therefore, pH adjustment should be carefully aligned with the individual pKa values of the fluoroquinolones under study. Variations in pH also influence electrostatic interactions between the analyte and the solvent system, thereby affecting extraction behavior and overall chromatographic performance. As the ionization state of an analyte changes, its retention behavior also changes. Ionizable analytes are highly sensitive to variations in the pH of the mobile phase, and even small pH fluctuations can result in significant changes in retention time. Changes in mobile phase pH also influence peak symmetry and width. When the pH of the mobile phase is close to the pKa of the analyte, both ionized and non-ionized forms may be present simultaneously, which can lead to peak splitting or shouldering. Under such conditions, even minor pH variations can have a pronounced effect on separation selectivity, particularly in the presence of structurally similar compounds.

### 6.4. Regulations Regarding the Maximum Residue Limit and Improvement of a Method Sensitivity

Maximum residue limit (MRL) is the highest concentration of a medicinal residue that is considered acceptable in food derived from an animal treated with that medicine. Residues of pharmacologically active substances include all pharmacologically active compounds, encompassing active substances, excipients, degradation products, and their metabolites that remain in food of animal origin. MRLs for pharmacologically active substances are established by appropriate regulatory authorities; for example, in the European Union, by the European Medicines Agency (EMA) through its Committee for Medicinal Products for Veterinary Use (CVMP), as well as by the Medicines and Healthcare products Regulatory Agency (MHRA). However, individual Member States may introduce their own MRLs. In Poland, FQLs may be applied to all animals except laying hens. The withdrawal period for edible tissues ranges from 3 to 10 days, depending on the animal species and the medicinal product used. Residues, for example ENR, in pig and chicken tissues may remain at high levels even 12 days after the end of treatment. Detailed data on MRLs for individual antibiotics in animal tissues, plants, and food of plant or animal origin can be found in the current regulations of the Minister of Agriculture, similarly to those in other Member States. New procedures should ensure the determination of drug residues at levels below the MRLs required for a given market. These methods should be reliable and compliant with the standards of accredited laboratories. Comprehensive information on the detected antibiotic and its concentration requires the use of appropriate analytical techniques, most commonly liquid chromatography. These methods should enable the detection and quantification of both the parent compound and potentially harmful metabolites. In the case of metabolite determination, analytical requirements are more stringent due to their presence at much lower concentrations than the parent substance. It is worth emphasizing that the lack of metabolite monitoring may complicate or obscure the interpretation of analytical results. Furthermore, due to the potential structural similarity of the metabolite to the parent substance, co-elution/co-migration may occur.

Most analytical methods aim to increase their sensitivity and reduce the LOD and LOQ. These objectives can be achieved in various ways, such as by using extraction methods. To assess the degree of sensitivity enhancement, enrichment or enhancement factors (EF) are commonly calculated in HPLC methods, while sensitivity enhancement factors (SEF) are typically calculated in CE methods. Some authors have reported these values in their studies [30,61,63,65,68,78,79,80,82]. EFs are calculated as the ratio between the slopes of the calibration curves obtained with and without the extraction method. EFs ranged from 7 to 20 for the 3D device coated with Zn/Co-zeolitic imidazole framework-derived carbon [30], from 43.4 to 45.6 for the graphene oxide/metal–organic framework-74/Fe_3_O_4_/polytyramine [61], from 29.1 to 43.9 for Fe_3_O_4_/multi-walled carbon nanotubes/ionic liquid [63], from 48 to 83 for vortex-assisted DLLM [65], and from 115.5 to 127.3 for the covalent organic framework/1,3,5-triformylphloroglucinol/p-phenylenediamine/Fe_3_O_4_ [68]. SEFs were estimated in several CE-UV procedures to characterize the degree of concentration of the analyte with the use of the developed method. The extraction efficiency is determined by the comparison of the size of the analyte peak obtained using the method with the extraction step and the analyte peak without extraction step. SEFs ranged from 103.7 to 127.4 for the C-18 modified MPs extraction method [80], from 531 to 858 for the SO-DLLME-DES-BE extraction method [79], and from 142.4 to 216.0 for the LLE extraction method [82].

## 7. Conclusions

The determination of antibiotics, including FQLs, in environmental and biological samples is essential for controlling the residues of these compounds in food and the environment, preventing drug resistance, and protecting public health. In most cases, the scale of the problems and challenges associated with FQL determination depends on the type of matrix used for analysis. Many analytical techniques, such as HPLC, CE, and electroanalysis, can be used to monitor FQL concentrations in more or less complex matrices. The rapid development of analytical methods aims to improve the sensitivity, selectivity, and speed of detection of these compounds in trace amounts. A key step in the precise determination of FQLs is effective sample preparation. The use of various extraction methods, including LLE and SPE, along with their miniaturized versions, allows for the selective removal of contaminants and the concentration of analytes from biological and environmental samples. A novel development is the use of nanomaterials (e.g., modified magnetic nanoparticles) to increase extraction efficiency and method sensitivity. Over the last five years, there has been a surge in multifaceted research into FQL assay methods. Methods based on HPLC supported by FL or MS detection remain the most common and accurate. Electrochemical biosensors are also rapidly gaining importance, primarily due to their practical advantages [84]. Simultaneously, the development of sample preparation techniques using nanomaterials is enhancing the sensitivity and efficiency of assays. These innovations are crucial, given the global challenges related to antibiotic resistance and the environmental monitoring of FQLs. A wealth of valuable information on methods for determining FQLs, including the sample preparation techniques used, can also be found in excellent review articles from recent years [84,85,86,87,88].

## Figures and Tables

**Table 1:** CE and HPLC methods for determination of fluoroquinolones (2020–2025).

Method	Sample	Analyte	Sample Preparation	LOD	LOQ	Precision	Accuracy	Run Time	Ref.
Electrophoretic procedures									
CE-ECL	Fish	CIP, ENR, NOR, PEF, OFL, FLE, LOM	SPME	0.4–0.8 ng/g	0.7–1.4 ng/g	not reported	89.2–110.4%	~10 min	[76]
CE-ECL	Fish	CIP, ENR, NOR, PEF	QuEChERS extraction	2.7–88 ng/g	54–880 ng/g	1.6–2.6%	not reported	~13 min	[77]
CE-ECL	Fish	CIP, ENR, OFL, NOR	Extraction, AuNP	6.6–16.5 ng/g	16.5–29.8 ng/g	not reported	not reported	~16 min	[78]
MEKC-UV	Milk, honey, water	SPA, GAT, ENR, CIP, LOM, LEV	SO-DLLME-DES-BE	6–10 μg/L	20–30 μg/L	2.1–6.5%	87.8–114.0%	~22 min	[79]
CE-UV	Meat	CIP, OFL	SPE, C-18 modified MPs	13.0 ng/g	49 ng/g	3.2–11.2%	93.0–99.5%	~10 min	[80]
CE-UV	Animal tissue	CIP, OFL	LLE	33.1–108.3 ng/g	99.3–288.8 ng/g	1.0–11.8%	90.4–104.6%	~15 min	[81]
CE-UV	Human urine	CIP, OFL	LLE	3.61–16.5 ng/mL	16.5–66.2 ng/mL	1.4–7.8%	86.4–102.6%	~7 min	[82]
Chromatographic procedures									
HPLC-FL	Environmental, aquaculture, and tap water	CIP, ENR	SPE, CMC@Co-MIP	0.15–0.21 μg/L	1.0 μg/L	<13%	64.9–102.3%	~18 min	[29]
HPLC-FL	Sewage	NOR, CIP, ENR, DIF, DAN	SPE, Zn/Co-ZIF-derived carbon	0.003–0.009 μg/L	0.01–0.03 μg/L	1.5–5.7%	91.0–104.0%	~22 min	[30]
HPLC-FL	Human urine and plasma	LEV, CIP, MOX, GEM	ACN deproteinization	26.93–35.63 μg/L	100 μg/L	0.4–8.1%	89.6–111.9%	~7.5 min	[31]
HPLC-FL	Animal urine	MAR, CIP, DAN, ENR, FLU	ACN and MeOH extraction	3.0–199.0 μg/L	9.0–664.0 μg/L	1.8–5.5%	35.0–102.0%	~12 min	[32]
UPLC-FL	Chicken	DAN, LOM, MAR, OFL, DIF, CIP, ENR, SAR	SPE, MIMs	0.2–0.8 ng/g	0.7–2.8 ng/g	4.6–17.1%	71.9–96.8%	~11 min	[33]
HPLC-FL	Human plasma	DEL	LLE	50.0 μg/L	100.0 μg/L	1.7–7.4%	94.0–103.0%	~12 min	[34]
UPLC-FL	Poultry eggs	CIP, ENR	LLE	0.1–0.5 ng/g	0.3–1.5 ng/g	2.0–6.2%	85.5–95.6%	~9 min	[35]
HPLC-FL	River water	NOR, LEV, CIP, DAN, ENR, LOM, SAR	On-line SPE, MIP	0.0001–0.0007 μg/L	0.0004–0.0022 μg/L	2.8–8.4%	66.0–101.0%	~60 min	[36]
HPLC-FL	Sewage	OFL, FLE, NOR	DLLME	0.3 μg/L	1 μg/L	<9%	>81%	~14 min	[37]
LC-MS/MS	Plasma	CIP	Ultrafiltration	not reported	50.0 ng/mL	1.3–4.9%	97.8–102.0%	~3 min	[38]
UHPLC-MS/MS	Plasma	CIP, LEV, PAZ	ACN and MeOH extraction	not reported	200.0–10,000 ng/mL	3.1–13.8%	92.7–108.3%	~7 min	[39]
LC-MS/MS	Wastewaters	CIP, ENR, LOM, OFL, NOR, MOX, PRU	SPE	0.0067–0.059 μg/L	0.022–0.197 μg/L	7.2–18.1%	80.3–92.9%	~4 min	[40]
HPLC-MS	Food	ENR, CIP, NOR, PEF, OFL	Dispersive solid-phase extraction, TFP-DABA MNS	not reported	0.1–0.2 ng/g	4.1–6.8%	94.5–105.8%	~12 min	[41]
UHPLC-HRMS	Fish	ENR, CIP	ACN extraction	0.1 ng/g	1.0 ng/g	3.4–12.8%	67.5–98.7%	~14 min	[42]
LC-MS/MS	Chicken manure, swine manure, poultry feed and soil	FLU, LEV, OFL, CIP, NOR, ENR, DAN, LOM, SAR	ACN and MeOH extraction	0.01–34.3 ng/g	0.03–115 ng/g	4.0–31.0%	60.0–143.0%	~20 min	[43]
LC-MS/MS	Food	CIP, DAN, DIF, ENR, FLU, NOR, SAR	Pressurized liquid extraction	25.0 ng/g	75.0 ng/g	8.7–61.4%	90–166.0%	~15 min	[44]
LC-MS/MS	Plasma	MOX, LEV	Deproteinization, TCA in ACN/MeOH	not reported	100.0–400.0 ng/mL	1.9–14.9%	91.4–109.7%	~10.5 min	[45]
UPLC-MS/MS	Fish	CIP, ENR, MAR, NOR, OFL	Homogenization with ethyl acetate and EDTA, SPE	0.05–0.25 ng/g	0.5–0.8 ng/g	4.9–12.1%	88.3–99.1%	~20 min	[46]
UPLC-MS/MS	Water	ENR, CIP, OFL, NOR, PEF, LOM, DAN, FLE, SAR, SPA, DIF, ENO	SPE	0.00002–0.0001 μg/L	0.00006 μg/L	0.9–13.2%	53.7–130.4%	~7 min	[47]
LC-MS/MS	Aqueous humor samples	LOM	LLE, diethyl ether	0.268 μg/L	0.894 μg/L	0.6–1.8%	98.1–101.9%	~7 min	[48]
LC-MS/MS	Sewage	CIP, NOR, OFL	QuEChERS extraction	0.02–2.77 ng/g	0.05–8.38 ng/g	4.0–7.0%	26.0–162.0%	~26 min	[49]
LC-MS/MS	Honeys	MAR, ENR	Extraction	0.44–0.75 ng/g	1.48–2.49 ng/g	1.0–1.3%	85.0–90.0%	~16 min	[50]
LC-HRMS/MS	Chicken liver	DAN, ENR	DLLME	0.665–0.744 ng/g	2.172–2.579 ng/g	3.0–8.8%	83.5–98.5%	~8 min	[51]
LC-MS	Fish	FLE, OFL, NOR, ENO, CIP, ENR, LOM, DAN, ORB, DIF, SAR, SPA, PEF	Homogenization with ethyl acetate and extraction with ACN	0.1–0.25 ng/g	0.4–1 ng/g	1.2–7.1%	67.7–112.8%	~20 min	[52]
HPLC-MS/MS	Plasma	GEM	Protein precipitation	not reported	5.0 ng/mL	1.7–10.7%	92.5–109.0%	~3 min	[53]
UPLC-MS	Chicken	ENR, CIP	Extraction	0.024–0.025 ng/g	0.080–0.085 ng/g	0.1–3.8%	93.1–103.0%	20–50 min	[54]
HPLC-MS/MS	Fish, shrimp	CIP	Liquid extraction (0.1% formic acid in MeOH)	1.279 ng/g	3.837 ng/g	0.6–2.6%	91.8–95.4%	~12 min	[55]
LC-MS/MS	Fish, shrimp	OFL, NOR, LOM, PEF	ACN extraction and SPE	0.12–0.59 ng/g	0.40–1.96 ng/g	1.9–2.6%	77.9–122.2%	~35 min	[56]
UPLC-MS/MS	Surface waters	CIP, OFL, NOR	SPE	1–2 μg/L	5 μg/L	not reported	71.0–125.0%	~16 min	[57]
HPLC-MS	Agricultural soils and sewage sludge	NOR, ENR, CIP	Extraction with MeOH and next d-SPE	0.05–0.06 ng/g	0.50 ng/g	1.6–14.0%	80.1–101.0%	~20 min	[58]
RP-HPLC-UV	Pharmaceutical formulations	MOX	Powdering and dissolving in MeOH	80.0 μg/L	243.0 μg/L	1.7–2.0%	99.0–100.5%	~4 min	[59]
HPLC-UV	Urine	LEV	SPE	22.0 ng/mL	73.3 ng/mL	1.1–5.3%	86.2–100.0%	~15 min	[60]
HPLC-UV	Chicken eggs	OFL, CIP, LOM, ENR, SPA	Extraction, MD-µ-SPE on GO/MOF-74/Fe_3_O_4_/PTy	0.1–0.4 μg/L	0.5–1.5 μg/L	3.3–7.2%	91.0–106.8%	~24 min	[61]
HPLC-UV	Drugs	CIP, MOX, NOR, OFL, PEF	Powdering and dissolving in water	10–25 μg/L	51–86 μg/L	0.8–1.0%	99.6–100.1%	~24 min	[62]
HPLC-UV	Animal urine	MAR, CIP, DAN, ENR, FLU	ACN and MeOH extraction	31.0–93.0 ng/mL	103.0–311.0 ng/mL	0.3–2.7%	35.7–100.0%	~12 min	[32]
HPLC-UV	Food	NOR, CIP, LOM, ENR	Magnetic solid phase extraction, Fe_3_O_4_/MWCNTs/IL	0.33–0.78 ng/mL	1.09–2.60 ng/mL	0.8–5.7%	85.2–105.9%	~14 min	[63]
HPLC-UV	Water	ENO, OFL, NOR, CIP, ENR	Magnetic solid phase extraction, Fe_3_O_4_@MON- NH_2_@CM-β-CD	1.4–2.3 μg/L	4.6–7.6 μg/L	0.1–8.4%	93.1–116.2%	~20 min	[64]
HPLC-UV	Environmental samples	CIP, DAN, ENR	Vortex assisted DLLM	0.00063–0.0012 μg/L	0.0021–0.004 μg/L	1.1–5.1%	99.0–108.0%	~10 min	[65]
HPLC-UV	Eye drops	OFL, GAT	Dilution with MeOH	387.0–698.0 μg/L	1434–2117 μg/L	0.5–1.0%	100.6–100.8%	~8 min	[66]
HPLC-UV	Chicken livers	NOR, SAR, DAN, CIP, ENR, DIF	Hydrophobic extraction by ionic and non-ionic surfactants	5–23 ng/g	15–78 ng/g	4.2–16.6%	61.0–104.0%	~14 min	[67]
HPLC-UV	Milk	ENO, FLE, OFL, NOR, PEF, LOM	Magnetic covalent organic framework extraction, COF(TpPa-1)@Fe_3_O_4_	0.05–0.20 μg/L	0.19–0.71 μg/L	3.5–4.7%	90.4–101.2%	~40 min	[68]
HPLC-UV	Drugs	ENO, NOR, CIP, LEV, MOX, ENR, SPA, MAR	Powdering and dissolving in water, NaOH or EtOH	not reported	15.0 μg/mL	0.1–1.9%	98.0–101.6%	~40 min	[69]
HPLC-DAD	Drugs	ENR	Extraction with ACN and dilution	96.0 μg/L	250.0 μg/L	<0.8%	99.8%	~6 min	[70]
LC-UV	Pharmaceutical formulation	LEV	Dissolving	not reported	1250.0 μg/L	<2%	96.6–99.5%	~10 min	[71]
HPLC-UV	Wastewater	CIP, NOR, OFL	QuEChERS	4.1–18.5 μg/L	13.5–61.6 μg/L	0.1–19.4%	38.0–120.0%	~5 min	[72]
HPLC-UV	Drug mixture	LEV	Dissolving drug in buffer	0.050 μg/L	0.320 μg/L	0.02–0.80%	99.1–100.3%	~7 min	[73]
HPLC-UV	Pharmaceutical formulations	MOX, LEV, GEM	Powdering and dissolving in EtOH	20.0–40.0 μg/L	80.0–140.0 μg/L	not reported	97.3–101.0%	~12 min	[74]
UHPLC-PDA	Human plasma	LEV, LOM, ENR, CIP, GAT, SPA, SAR, DAN	SPE	1.0 ng/mL	5.0 ng/mL	<5.2%	~98.9%	~7 min	[75]

Salting out-assisted dispersive liquid–liquid microextraction based on deep eutectic solvent—(SO-DLLME-DES-BE); Co^2+^-mediated paper-based MIPs enrichment chip—(CMC@Co-MIP); 3D device coated with Zn/Co-zeolitic imidazole framework-derived carbon—(Zn/Co-ZIF-derived carbon); the mixed-mode magnetic sulfonated covalent organic framework composites—(TFP-DABA MNS); magnetic dispersive micro-solid phase extraction—(MD-µ-SPE); graphene oxide/metal–organic framework-74/Fe_3_O_4_/polytyramine—(GO/MOF-74/Fe_3_O_4_/PTy); Fe_3_O_4_/multi-walled carbon nanotubes/ionic liquid—(Fe_3_O_4_/MWCNTs/IL); Fe_3_O_4_/microporous organic network-NH_2_/carboxymethylated-β-CD—(Fe_3_O_4_@MON-NH_2_@CM-β-CD); covalent organic framework/1,3,5-Triformylphloroglucinol/p-phenylenediamine/Fe_3_O_4_—(COF(TpPa-1)@Fe_3_O_4_); dispersive solid-phase extraction—(d-SPE).

## Data Availability

Not applicable.

## Abbreviations

The following abbreviations are used in this manuscript:

ACN	Acetonitrile
CE	Capillary electrophoresis
CIP	Ciprofloxacin
DAN	Danofloxacin
DEL	Delafloxacin
DIF	Difloxacin
ECL	Electrochemiluminescent detection
ENO	Enoxacin
ENR	Enrofloxacin
EtOH	Ethanol
FL	Fluorescence detection
FLE	Fleroxacin
FLU	Flumequine
FQL	Fluoroquinolone
GAT	Gatifloxacin
GEM	Gemifloxacin
HPLC	High performance liquid chromatography
LEV	Levofloxacin
LOM	Lomefloxacin
MAR	Marbofloxacin
MeOH	Methanol
MOX	Moxifloxacin
MS	Mass spectrometry
NOR	Norfloxacin
OFL	Ofloxacin
ORB	Orbifloxacin
PAZ	Pazufloxacin
PEF	Pefloxacin
PRU	Prulifloxacin
Ru(bpy)_3_^2+^	Tris (2,2′-bipyridyl) ruthenium (II)
SAR	Sarafloxacin
SPA	Sparfloxacin
UV	Ultraviolet detection
